# Spatial induction of genes encoding secreted proteins in micro-colonies of *Aspergillus niger*

**DOI:** 10.1038/s41598-020-58535-0

**Published:** 2020-01-30

**Authors:** Martin Tegelaar, David Aerts, Wieke R. Teertstra, Han A. B. Wösten

**Affiliations:** Microbiology, Utrecht University, Padualaan 8, 3584 CH Utrecht, The Netherlands

**Keywords:** Industrial microbiology, Applied microbiology

## Abstract

*Aspergillus niger* is used by the industry to produce enzymes and metabolites such as citric acid. In liquid cultures, it can grow as a dispersed mycelium or as micro-colonies with a width in the micrometer to millimeter range. Here, it was assessed whether expression of genes encoding secreted enzymes depends on mycelium morphology. To this end, expression of the reporter gene *gfp* from the promoters of the glucoamylase gene *glaA*, the feruloyl esterase gene *faeA* and the α-glucuronidase gene *aguA* was causally related to micro-colony size within a liquid shaken culture. Data could be fitted by hyperbolic functions, implying that the genes encoding these secreted proteins are expressed in a shell at the periphery of the micro-colony. The presence of such a shell was confirmed by confocal microscopy. Modelling predicted that the width of these zones was 13 to 156 µm depending on growth medium and micro-colony diameter. Together, data indicate that the highest productive micro-colonies are those colonies that have a radius ≤ the width of the peripheral expression zone.

## Introduction

Filamentous fungi form mycelia that consist of a network of hyphae that grow at their tips and that branch sub-apically. Mycelia of aspergilli can reach a diameter in the sub-millimeter scale (micro-colonies) to centimeter scale (macro-colonies) depending on the size and the composition of the solid substrate^1^. For instance, *Aspergillus* forms micro-colonies on wheat kernels, whereas macro-colonies can be formed on fruits or bulbs of plants. In liquid shaken cultures, mycelium of *Aspergillus* grows dispersed, as micro-colonies, or in an intermediate state called clumps^1^. Dispersed mycelium consists of small networks of hyphae, while micro-colonies, also known as pellets, consist of a clear central and outer zone^2^. Notably, micro-colonies of *Aspergillus niger* produce more citric acid when compared to dispersed mycelium^3,4^. Why micro-colonies are more productive is not yet clear. It may be caused by the effect of the fungal morphology on the viscosity of the medium being high and low during dispersed growth and growth as micro-colonies, respectively^5^. At the same time, availability of oxygen and nutrients may impact productivity. Diffusion of these compounds would be sufficient in the case of dispersed mycelium while it would be limiting in the center of micro-colonies^6^.

So far, it is not known whether morphology of the mycelium affects production of secreted proteins. It was shown that micro-colonies within a liquid shaken culture are heterogeneous with respect to size and gene expression^2^. However, the relation between colony size and expression of genes encoding genes was not assessed. Therefore, we here studied whether micro-colony diameter affects expression of genes encoding secreted proteins in *A. niger*. To this end, the highly expressed genes encoding feruloyl esterase FaeA^7,8^, α-glucuronidase AguA^7,9^ and glucoamylase GlaA^7,10^ were used as model genes of xylanolytic (FaeA, AguA) and amylolytic (GlaA) activity. Data show that these genes are expressed in the outer part of micro-colonies only. Results imply that small micro-colonies of *A. niger* produce more secreted proteins per biomass than large micro-colonies.

## Results

### Effect of mycelium transfer on micro-colony size

**Figure 1:**
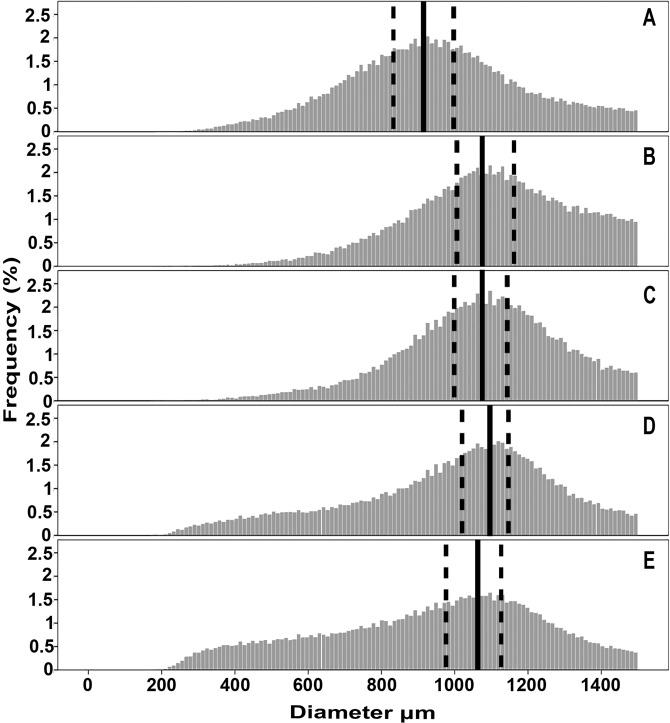
Size distribution of N402 micro-colonies that were not transferred to fresh medium (**A**) or were transferred from CMX to CMX (**B**), CMM (**C**), MMX (**D**) or MMM (**E**). Solid black lines flanked by dashed black lines represent the modes of the micro-colony populations and their 95% confidence intervals, respectively.

**Figure 2:**
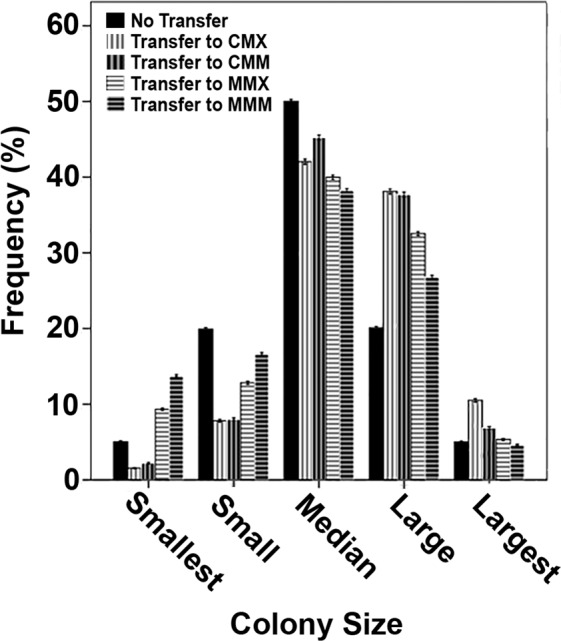
Proportions of size categories of N402 micro-colonies that were not transferred to fresh medium or were transferred from CMX to CMX, CMM, MMX or MMM. Error bars represent 95% confidence intervals.

Wild-type strain N402 was grown in CMX (complete medium with xylose) for 16 h followed by a transfer to either CMX, CMM (complete medium with maltose), MMX (minimal medium with xylose) or MMM (minimal medium with maltose). Biomass of micro-colonies increased during a 4 h growth period from 0.6 g to 0.98, 0.84, 0.90 and 0.76 g, respectively. This was accompanied by an increase in mean micro-colony diameter from 948 µm to 1091, 1065 and 987 µm, in the case of CMX, CMM and MMX respectively. In contrast, mean micro-colony diameter was reduced by 19 µm after transfer to MMM. The size distribution of micro-colonies was also altered after transfer to the different media. In all cases, the mode was shifted to a higher diameter by ~163 µm (Fig. 1). However, a higher number of small micro-colonies were found as well upon transfer to MMX and MMM (Fig. 1D,E). This is illustrated by plotting the frequency of micro-colonies with diameters < 2.5^th^, > 2.5^th^ < 25^th^, > 25^th^ < 75^th^, > 75^th^ < 97.5^th^ and > 97.5^th^ percentile (Fig. 2). Transfer from CMX to MMX resulted in an increased proportion of micro-colonies within the smallest 2.5% of diameters as well as the largest 25% of diameters. At the same time, the frequency of micro-colonies of median sized micro-colonies was reduced. This shift was exacerbated when CMX grown micro-colonies were transferred to MMM, while transfer of micro-colonies from CMX to CMX only led to an increase in proportion of the micro-colonies within the largest 25% diameters and a decrease in proportion of micro-colonies within the median and smaller 25% of diameters. These findings were confirmed by multinomial logistic regression (data not shown). Together, data show that transfer to fresh medium promotes radial growth of N402 micro-colonies and, depending on the medium, can also result in partial micro-colony fragmentation.

**Figure 3:**
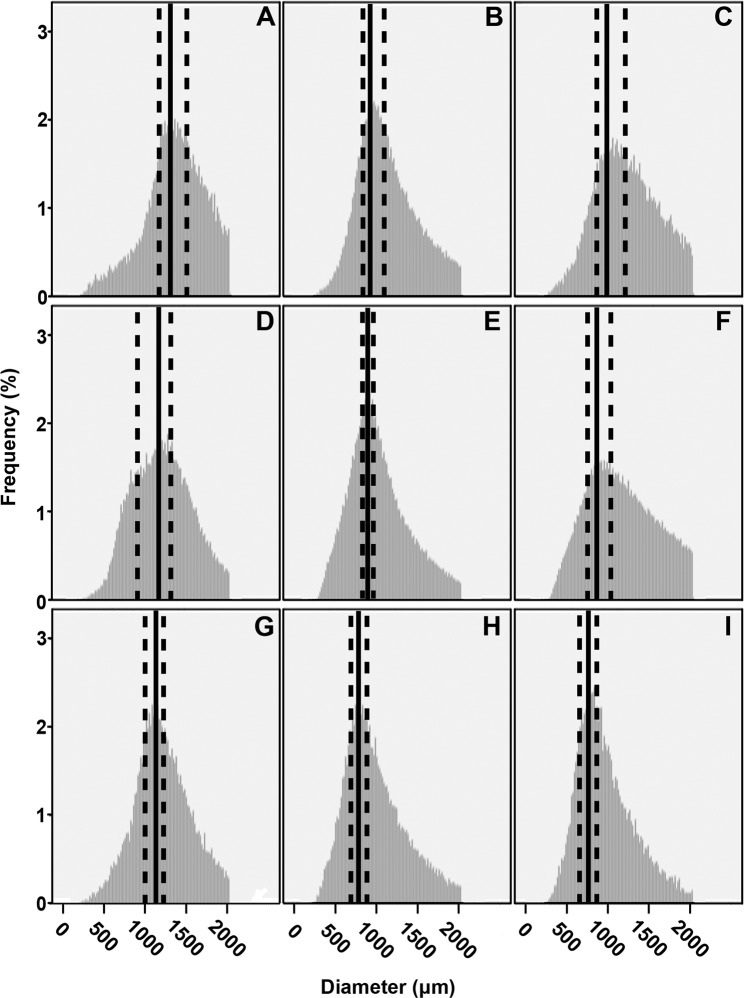
Size distribution of *glaA::GFP* (**A,D,G**)*, aguA::GFP* (**B,E,H**) and *faeA::GFP* (**C,F,I**) micro-colonies that were or were not transferred from repressing CM (**A**–**C**) to inducing MM (**D**–**F**) or inducing CM (**G**–**I**). Solid black lines flanked by dashed black lines represent the modes and their 95% confidence intervals for the micro-colony populations, respectively.

**Figure 4:**
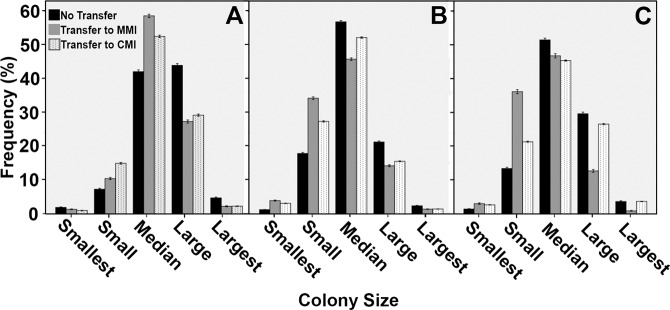
Proportions of size categories of *glaA::GFP* (**A**)*, aguA::GFP* (**B**) and *faeA::GFP* (**C**) micro-colonies that were not or were transferred from repressing CM to inducing MM or CM. Error bars represent 95% confidence intervals.

Like wild-type N402, biomass of strains expressing *gfp* from the *glaA*, *faeA* or *aguA* promoter increased upon transfer of 16 h old mycelium of non-inducing CM to inducing MM or CM. Biomass of the *glaA::GFP*, *aguA::GFP* and *faeA::GFP* strains had increased in MM from 0.71 to 0.78 g, 0.73 to 0.83 g and 0.73 to 0.93 g during a 4 h period, respectively. When transferred to inducing CM, biomass of the *glaA::GFP*, *aguA::GFP* and *faeA::GFP* strains had increased to 0.85, 1.47 and 1.49 g, respectively. Also in these strains, fragmentation occurred when mycelium was transferred to fresh medium (Fig. 3). In fact, mean micro-colony diameter of *glaA::GFP* (1361 µm), *aguA::GFP* (1142 µm) and *faeA::GFP* (1230 µm) was reduced by 140, 145 and 250 µm, respectively, after a 4 h transfer to inducing MM. This reduction was 180, 130 and 79 µm for *glaA::GFP*, *aguA::GFP* and *faeA::GFP*, respectively, after transfer to CM. Transfer of reporter strains to inducing MM or CM coincided with a decrease in the proportion of micro-colonies within the largest 25% of diameters and an increase in proportion of micro-colonies within the smallest 25% of diameters (Fig. 4). Proportions of median sized micro-colonies also decreased for the *aguA::GFP* and *faeA::GFP* strains. In contrast, the *glaA::GFP* strain showed a marked increase in the proportion of median sized micro-colonies. Increase and decrease of proportions of size categories were of lesser magnitude after transfer of fluorescent strains to inducing CM. Diameters of small, median and large micro-colonies of the *faeA::GFP* and *aguA::GFP* strains had reduced after transfer to inducing MM. For *glaA::GFP*, diameter of median and large microcolonies had also reduced after transfer to inducing MM. For the *faeA::GFP* and *aguA::GFP* strains transferred to inducing CM the smallest 2.5% of micro-colonies had increased in diameter, while small and median micro-colonies had reduced diameter. Large micro-colonies of *aguA::GFP* were also reduced in diameter, while large micro-colonies of *faeA::GFP* showed a slight increase in diameter. Micro-colony diameter of the *glaA::GFP* strain was reduced for median and large sized colonies but had increased for small micro-colonies. These findings were confirmed by multinomial logistic regression (data not shown). Together, biomass of the reporter strains increased after transfer to inducing medium but size of micro-colonies was reduced by micro-colony fragmentation.

### Relation between micro-colony size and expression of genes encoding secreted proteins

**Figure 5:**
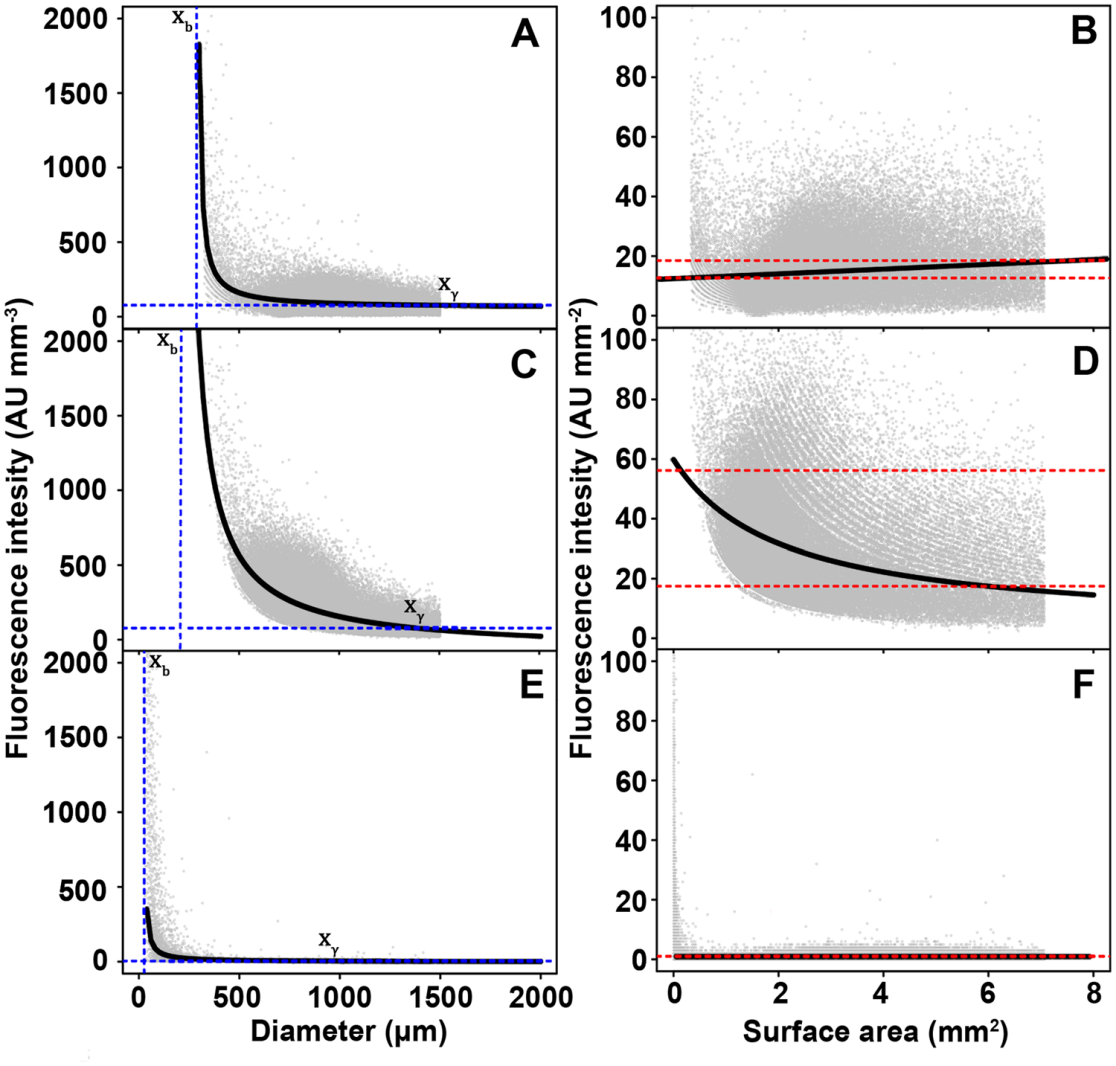
Relation between fluorescence per volume and pellet diameter (**A,C,E**) and fluorescence per surface area and surface area (**B,D,F**) of strains expressing *gfp* from the *glaA* (**A**,**B**), *aguA* (**C**,**D**) and *faeA* (**E**,**F**) promoter. Gray circles represent individual micro-colonies. The solid line represents the best fit as determined by quantile regression of the median. Horizonal blue dashed lines represent *FV*^−*1*^ at steady state and vertical blue dashed lines represent the diameter where fluorescence intensity is maximal (*x*_*b*_). The intersect between *FV*^*−1*^ at steady state and the lower 95% confidence limit of the best fit is denoted by *x*_*γ*_. The red dashed lines (**D**–**F**) represent minimal and maximal fluorescence intensity mm^−2^ as predicted by EQ3.

The relation between micro-colony diameter and GFP fluorescence intensity was studied in the reporter strains *glaA::GFP*, *aguA::GFP* and *faeA::GFP*. As expected, non-induced strains or the wild-type strain N402 showed low or no fluorescence (Supplemental Fig. 1E). Total fluorescence intensity of reporter strains transferred to inducing medium increased quadratically with increasing diameter (Supplemental Fig. 2). Consequently, fluorescence intensity per micro-colony volume (*FV*^*−1*^) per diameter was best fitted by a hyperbolic function (Fig. 5A–E, Supplemental Fig. 3A–E). In this function, *FV*^*−1*^ is maximal at the vertical asymptote *x*_*b*_ of the hyperbolic function, while with increasing diameter *x*, *FV*^*−1*^ decreases. Maximal *FV*^*−1*^ of *glaA::GFP, aguA::GFP* and *faeA::GFP* micro-colonies that had been induced in MM was found at a diameter of 288, 211 and 26 µm, respectively (Table 1). When strains were induced in CM maximal *FV*^*−1*^ was found at a diameter of 169 and 216 µm for *aguA::GFP* and *faeA::GFP*, respectively. Conservative selection parameters derived from micro-colony profiles could lead to micro-colonies being omitted from analysis unintentionally. Therefore, the diameter where *FV*^*−1*^ is maximal could be smaller in reality. This goes particularly for the CMM induced *glaA::GFP* strain as *a* is equal to 0, making *b* an arbitrary number intersecting *γ* with a minimum concurrent with the smallest recorded diameter. A hyperbolic function also has a horizontal asymptote (*γ*) that approaches a steady state. For *glaA::GFP, aguA::GFP* and *faeA::GFP* induced in MM this steady state was reached at 1556, 1381 and 954 µm, respectively. The horizontal asymptote was reached at a diameter of 1254 and 1532 when *aguA::GFP and faeA::GFP* were induced in CM (Table 1). Slopes did not converge to a steady state in the case of CMM-induced *glaA::GFP*.

**Table 1:** Values of relevant parameters from models describing the relation between micro-colony size and expression of *gfp* from the *glaA, aguA* and *faeA* promoters.

Inducing medium	Promoter	x_b_ (µm)	x_γ_ (µm)	r_γ_ (µm)	r_γ_/0.5x_γ_ (%)
*MM*	*glaA*	288 (±8)	1556 (−80/+ 109)	156 (±0.8)	21.5 (±0.1)
	*aguA*	211 (±11)	1381 (−11/+ 15)	89 (±0.2)	8.7 (±0.02)
	*faeA*	26 (±0.3)	954 (−1.7/+ 2.7)	13 (±0.2)	2.7 (±0.02)
*CM*	*glaA*	—	—	—	—
	*aguA*	169 (±3)	1254 (±3)	37 (±0.3)	1 (±0.01)
	*faeA*	230 (±12)	1532 (±4)	31 (±2)	2 (±0.01)

Numbers between brackets indicate the 95% confidence interval. x_b_ is micro-colony diameter where fluorescence intensity per volume (FV^−1^) is maximal, x_γ_ represents the diameter where FV^−1^ remains constant when diameter increases, r_γ_ is the predicted radius of the fluorescent band at this steady state and r_γ_/0.5x_γ_ represents the percentage of the radius of the micro-colony that shows fluorescence.

**Figure 6:**
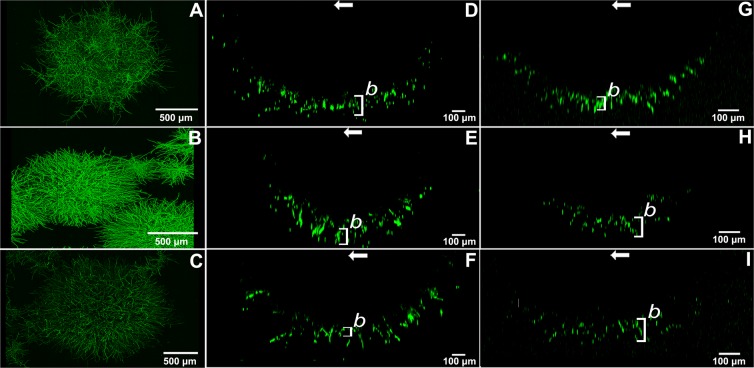
Maximum intensity Z projectionsremoved from the dataset because of confocal scanning images of the center of micro-colonies (**A–C**) and their respective YZ profiles from the center (**D–I**) of *glaA::GFP* (**D–G**), *aguA::GFP* (**E–H**) and *faeA::GFP* (**F–I**) that were induced in either MM (**D,E,F**) or CM (**G–I**). Arrows mark the center of the colony. Bars marked *b* denote the mean thickness of the fluorescent shell.

Increase in fluorescence intensity in the region between *x*_*b*_ and *x*_*γ*_ cannot be explained solely by increase in micro-colony volume when parameter α in Eq. 2 is shown not to be zero. This suggests that fluorescence is not evenly distributed over micro-colony volume but occurs in a concentric peripheral shell (Supplemental Fig. 4). Indeed, z-stacks of confocal laser scanning microscopy images showed a fluorescent concentric zone with a radius of 149, 128 and 63 µm for *glaA::GFP, aguA::GFP* and *faeA::GFP* when induced in MM and 69, 102 and 118 µm when induced in CM, respectively (Table 2, Fig. 6; Supplemental Movies 1–3). To determine whether the width of the concentric shells depends on micro-colony size, fluorescence intensity per surface area was modeled (Fig. 5B–F; Supplemental Fig. 3B–F). In the case of MM, change in width was < 10%. Modelling inferred that in the case of MM expression of *faeA* occurs in a relatively small concentric shell with a constant radius of 13 µm. In contrast, *glaA* is expressed in a concentric outer shell that increases in width from 144 to 157 µm as micro-colonies increase in diameter from 288 to 1556 µm. On the other hand, *aguA* was predicted to be expressed in a concentric outer shell with a width decreasing from 105 µm to 92 µm in the 210 to 1381 µm size range of micro-colonies. When reporter strains were induced in CM, *glaA* was predicted to be expressed in a concentric outer shell that increases with a square root relationship. On the other hand, *aguA* was predicted to be expressed in a concentric outer shell that has a width decreasing from 85 µm to 37 µm in the 169 to 1245 µm size range of micro-colonies. Moreover, *faeA* was predicted to be expressed in a concentric outer shell with a width decreasing from 85 µm to 31 µm in the 230 to 1532 µm size range of micro-colonies. Together, upon growth of the micro-colonies, the volume of the expression zone increases. At the same time its relative radius and volume decreases compared to the total diameter and volume of the pellet.

**Table 2:** Radius of the expression zones of *glaA-, aguA-*, and *faeA-*driven *gfp* expression as shown by confocal microscopy.

Inducing medium	Promoter	Fluorescent radius (µm)	Colony Diameter (µm)	Fluorescent radius (%)
*MM*	*glaA*	149 (±19)	1673 (±197)	17,8 (−4/+5)
	*aguA*	128 (±18)	1332 (±256)	19,2 (−5/+8)
	*faeA*	63 (±17)	1795 (±155)	7 (−2,4/+2,8)
*CM*	*glaA*	69 (±7)	1025 (±22)	6,7 (±0,6)
	*aguA*	102 (±16)	1045 (±43)	9,8 (±1,5)
	*faeA*	118 (±13)	1065 (±27)	11,1 (±1,2)

Numbers between brackets indicate the 95% confidence interval.

## Discussion

Morphology of the mycelium in liquid cultures such as bioreactors depends on various factors including inoculum size, surface composition of the inoculum, medium composition, and mixing conditions^1,7,11^. In addition, presence of molecules such as chelating agents or anionic polymers may affect morphology. Here, it was shown for the first time that transfer of mycelium to fresh medium, in particular when changing the carbon source, also affects the morphology of the mycelium by inducing its fragmentation. Future studies will assess the underlying mechanisms and whether the observed fragmentation can be used to optimize productivity in liquid cultures.

So far, it had not been established how secretion of proteins relates to the morphology of the mycelium. Driouch *et al*.^12^ showed that 400-µm-wide micro-colonies express the glucoamylase gene *glaA* throughout the mycelium while expression in mm-sized microcolonies is only observed at the outer periphery. However, this study made use of the presence or absence of titanate micro-particles to control morphology. Therefore, changes in spatial gene expression may have been the result of the addition of the micro-particles. Here, we did not compare two cultures with different composition but rather made use of the heterogeneity of micro-colony morphology within a liquid shaken culture. Expression of *glaA, faeA* and *aguA* per micro-colony volume generally decreased with increasing micro-colony size in a hyperbolic way, eventually approaching a constant expression per micro-colony volume. The hyperbolic relationship between colony diameter and fluorescence per volume is explained by expression taking place in a concentric shell at the periphery of the micro-colony. Expression in such a concentric shell was confirmed by fluorescence microscopy. When compared to confocal microscopy data models were reasonably accurate in predicting the fluorescent radius of *glaA* and *aguA* after transfer to inducing minimal medium; predicting the fluorescent radius of *glaA::GFP* within its 95% confidence interval and underpredicting the fluorescent radius for *aguA::GFP* by 16%. Yet, the expression zone of *faeA* deviated by 72%. For reporter strains transferred to inducing complete medium the fluorescent radius of the *glaA::GFP* strain could not be predicted because there were only 3% entirely fluorescent colonies in the culture. The fluorescent radius of *aguA::GFP* and *faeA::GFP* was under-predicted by 64% and 74%, respectively. Underprediction of the expression zone of *faeA* may be caused by the relatively low expression of this gene, while underprediction of expression zones may also be caused by selection of micro-colonies in the confocal analysis that had not yet reached their steady state (i.e. they were too small). Particle analysis is therefore the preferred tool to assess expression zones because it takes into account all micro-colonies sizes in a culture instead of analysing single micro-colonies.

GFP expression was also found at the outer part of macro-colonies^13^. In contrast, the spatial expression in micro-colonies resulting from the *aguA* and *faeA* promoters was not in accordance with those in macro-colonies^13^. These genes were found to be expressed throughout the macro-colony and the colony centre, respectively. The different spatial expression patterns in micro- and macro-colonies may be explained by inducer penetration. Micro-colonies in liquid shaken cultures are 3D structures. Penetration of the inducer into the centre of the colony may be difficult. In contrast, macro-colonies grown on solid medium are near 2D and therefore the inducer in the underlying medium has access to all zones of the colony. However, this does not explain why the radius of the fluorescent concentric shell of the *faeA::GFP* strain is relatively small when compared to the *aguA::GFP* strain despite the fact that both genes are induced by xylose. Possibly, these genes respond to different concentrations of the inducer.

The fact that expression of the genes encoding secreted proteins occurs at the periphery of the micro-colony in a shell with a relatively constant width means that the volume of this shell decreases relatively to the total volume of the micro-colony when the micro-colony becomes larger. Thus, cultures with uniform small micro-colonies (diameters at *x*_*b*_) would be more productive than cultures with uniform large micro-colonies (diameters at) assuming similar biomass is formed per volume culture medium. Glucoamylase, α-glucuronidase and feruloyl esterase production would then be 54, 56 and 91% less efficient at steady state diameters compared to micro-colonies with diameters at *x*_*b*_ (i.e. diameter where expression per volume unit is maximal) if grown in minimal medium. In inducing complete medium, α-glucuronidase and feruloyl esterase production would be 61 and 65% less efficient at steady state diameters compared to micro-colonies with diameters at *x*_*b*_.

## Methods

### Strains and culture conditions

*Aspergillus niger* strains N402, UU-A005.4, AR9#2 and AV11#3 were used in this study (Table 3). The former strain was used as a control, while the latter three strains (called *faeA::GFP*, *glaA::GFP* and *aguA::GFP* in this chapter) express *gfp* from the *faeA*, *glaA* and *aguA* promoter, respectively. Strains were grown at 30 °C on minimal medium (MM^14^,) with 25 mM xylose and 1.5% agarose (MMXA). MMXA cultures were grown for three days, after which conidia were harvested using Saline-Tween (0.8% NaCl and 0.005% Tween-80). 250 ml liquid cultures were inoculated with 1.25*10^9^ freshly harvested conidia and grown at 200 rpm and 30 °C in 1 L Erlenmeyer flasks in complete medium (CM) (MM containing 0.5% yeast extract and 0.2% enzymatically hydrolyzed casein) supplemented with either 25 mM glucose (CMG; repressing *aguA* and *faeA* expression) or 25 mM xylose (CMX; repressing *glaA* expression). Mycelium was harvested after 16 h and washed twice with PBS. Ten g of biomass (wet weight) was transferred to CMX or MM with 25 mM xylose (MMX) (inducing *aguA* and *faeA*) or to CM or MM with 25 mM maltose (CMM, MMM) (inducing *glaA* expression). Biomass of cultures prior to transfer and after 4 h of growth in inducing medium was quantified after freeze drying.

**Table 3:** Strains used in this study.

Strain	Parental Strain	Genotype	Reference
N402	CBS 120.49	*cspA1, amdS*^*−*^	^17^
AR9#2	AB4.1 (derivative of N402)	pyrG^+^, sGFP(S65T) under regulation of the *A. niger glaA* promoter	^13^
AV11#3	AB4.1 (derivative of N402)	pyrG^+^, sGFP(S65T) under regulation of the *A. niger aguA* promoter	^13^
UU-A 005.4	NW249 (derivative of N402)	Nic-, leu- arg-, pyrG^+^, sGFP(S65T) under regulation of the *A. niger faeA* promoter	^13^

### Flow cytometry

Mycelium of liquid shaken cultures (biological triplicates) was fixed overnight at 4 °C in 4% paraformaldehyde in PBS, washed twice with PBS and taken up in 50 ml PBS supplemented with 150 mM glycine to quench autofluorescence. Diameter and fluorescence were analyzed using a BioSorter (Union Biometrica, Boston, MA, USA) equipped with a 2 mm nozzle (fluidics and optics core assembly (FOCA) 2000) and using a 488 nm solid state laser. At least 10000 particles per replica were analyzed for size and fluorescence measurements. Diameter of micro-colonies was determined from the time of flight (TOF) using $x=\frac{-b\pm \sqrt{{b}^{2}-4a\cdot -TOF}}{2a}({\rm{a}}=0,00215762,\,{\rm{b}}=5,433189436)$. This formula was inferred from the TOF of beads with diameters of 42 µm, 250 µm and 500 µm. Micro-colony volume (V) was calculated from the diameter assuming spherical morphology. To analyze the relation between micro-colony morphology and gene expression, particles with a TOF <165 and >13005 arbitrary units (AU) were removed from the dataset because they are outside the object size range of the FOCA (i.e. <30 µm and >1500 µm). In addition, particles were removed with fluorescence peaks <80 AU. Peaks >80 AU but with a fluorescence width <2000 AU were also removed. Finally, particles were removed that had an integrated density <25 AU for the green, red and yellow channels. The integrated green value (in AU) from the Biosorter was taken as a measure for fluorescence intensity (F). Micro-colonies of the non-fluorescent wild-type strain N402 were selected similarly but in this case particles were removed that had fluorescence peaks <100 AU. Additionally, integrated density of fluorescent signals was no longer a selection variable.

Differences in size distributions were quantified using two-sample Kolmogorov-Smirnov tests. Particles were distributed into five size categories based on diameter. Micro-colonies with diameters < 2.5^th^, > 2.5^th^ < 25^th^, > 25^th^ < 75^th^, > 75^th^ < 97.5^th^ and > 97.5^th^ percentile were designated smallest, small, median, large and largest, respectively. Differences in ratio between categories were analyzed using chi-square tests. Change of diameter after transfer to different media was described by resampling with 1000-fold replacement. For each resample the mode was recorded as the central tendency. The median of the modes and the 2.5^th^ and 97.5^th^ percentile provided an approximation of the mode and its confidence intervals. For biomass quantification, micro-colonies were not fixed, filtered using coffee filters, transferred to 50 ml falcon tubes and freeze-dried. Differences in dry weight were analyzed using Kruskal–Wallis H test.

### Modelling GFP fluorescence relative to micro-colony diameter

Median fluorescence intensity data of micro-colonies that had been transferred to inducing medium were modelled with the R package quantreg^15^ using non-linear quantile regression. Data pertaining to the fluorescence intensity per unit of volume (mm^3^) were best described by the non-linear hyperbolic functions Eqs. 1 and 2.

1 $$F{V}^{-1}=\frac{a}{x-b}+c$$

2 $$\frac{dF}{dV}=\frac{\alpha }{V}+\gamma $$

In these equations, *F* denotes fluorescence intensity and *V* micro-colony volume (mm^3^). In Eq. 1, *x* denotes micro-colony diameter (µm), a represents the maximal fluorescence intensity per volume, *c* denotes the arbitrary intercept of this function and *b* represents the location of the vertical asymptote (i.e. the diameter *x*_*b*_ of the micro-colony where fluorescence intensity per volume is maximal). In Eq. 2, α denotes the maximal fluorescence intensity per volume, while *γ* represents a steady state between increase in fluorescence intensity and increase in volume. The x coordinate *x*_*γ*_ of the intersect of the lower 95% confidence limit of Eq. 1 and *γ* gives the diameter of micro-colonies that have reached this steady state.

Parameters *x*_*b*_ and *x*_*γ*_ were transformed to their corresponding surface areas, *S*_*b*_ and *S*_*γ*_ (mm^2^) and used as input in EQ3 to determine if fluorescence intensity increases proportionally to micro-colony size. Using *S*_*b*_ and *S*_*γ*_ as input for *S*, EQ3 gives the minimal $(F{S}_{b}^{-1})$ and maximal $(F{S}_{\gamma }^{-1})$ observed fluorescence intensity per unit of surface area. Equation 3.1 was used for *glaA::GFP*, while Eq. 3.2 was used for *aguA::GFP* and *faeA::GFP*.

3.1 $${F}{{S}}_{({b};\gamma )}^{-1}=g{{S}}_{({b};\gamma )}+i$$

3.2 $${F}{{S}}_{({b};\gamma )}^{-1}=\frac{{{g}}_{0}}{{{S}}_{({b};\gamma )}-l}+i$$

in which *S* denotes surface area in mm^2^, while *i* represents the intercept, *g*_0_ the slope and *l* the arbitrary location of the vertical asymptote. In this equation, any difference between ${F}{{S}}_{\gamma }^{-1}$ and ${F}{{S}}_{{b}}^{-1}$ in fluorescence per surface area is due to changing fluorescence intensity within the micro-colony, change of the radius of a hypothesized fluorescent shell, and/or the change in volume of this fluorescent shell due to increase in micro-colony size. Equation 4 was used to rule out that the difference between ${F}{{S}}_{\gamma }^{-1}$ and ${F}{{S}}_{{b}}^{-1}$ is explained by the change in volume of the fluorescent shell. In this equation the change of fluorescence intensity per surface area is given by the derivative of *FS*^−1^: *f* ′(*S*).

4 $$\Delta {F}{{S}}_{{b}\gamma }^{-1}=\,{\int }_{{{\rm{S}}}_{{\rm{b}}}}^{{{\rm{S}}}_{\gamma }}f^{\prime} ({S})-{\int }_{{{V}}_{{{S}}_{{b}}}}^{{{V}}_{{{S}}_{\gamma }}}f^{\prime} ({{S}}_{{v}})$$

where ${V}_{{s}_{b}}$ and ${V}_{{s}_{\gamma }}$ represent the minimal and maximal volume of the fluorescent shell. For derivative *f* ′(*S*_*v*_) the variable *S* has been substituted by $\frac{{{S}}^{\frac{3}{2}}-{({S}-{{S}}_{{b}})}^{\frac{3}{2}}}{6\sqrt{\pi }}$ to describe the volume of the fluorescent shell. When $\Delta {F}{{S}}_{{b}\gamma }^{-1}$ does not equal 0, fluorescence intensity mm^−2^ can only be explained by change in width of the fluorescent shell in the z-axis or its fluorescence intensity. The surface and volume independent change of fluorescence intensity of the fluorescent shell is revealed by Eq. 5.1 and Eq. 5.2, which were used for *glaA::GFP* and *aguA::GFP* and *faeA::GFP*, respectively.

5.1 $${F}_{i}={\int }_{{S}_{b}}^{{S}_{\gamma }}\frac{\Delta F{S}_{b\gamma }^{-1}}{{S}_{\gamma }-{S}_{b}}S-\frac{\Delta F{S}_{b\gamma }^{-1}}{{S}_{\gamma }-{S}_{b}}{S}_{b}$$

5.2 $${F}_{i}={\int }_{{S}_{b}}^{{S}_{\gamma }}\frac{\frac{(F{S}^{-1}-F{S}_{b}^{-1})}{(S-{S}_{b})}\frac{(S-{S}_{\gamma })}{(F{S}^{-1}-F{S}_{\gamma }^{-1})}}{\frac{(\sqrt{g}+i+\Delta F{S}_{b\gamma }^{-1}-F{S}_{\gamma }^{-1})}{(\sqrt{g}+l-{S}_{b})}\frac{(\sqrt{g}+l-{S}_{\gamma })}{(\sqrt{g}+i-F{S}_{b}^{-1})}}-F{S}_{b}^{-1}$$

When *F*_*i*_ is substituted in Eq. 6, the hypothesized increase of fluorescent radius z is found.

6 $$\begin{array}{rcl}{V}_{s} & = & \frac{{F}_{i}+{\int }_{{S}_{b}}^{{S}_{\gamma }}F{S}_{(b:\gamma )}^{-1}\,}{m}\\ {V}_{{s}_{b}} & = & \frac{{\int }_{{S}_{b}}^{{S}_{\gamma }}F{S}_{(b:\gamma )}^{-1}}{m}\\ {V}_{s}\cdot {10}^{9} & = & \frac{1}{6}\pi ({x}^{3}-{(x-{x}_{b})}^{3})\\ {V}_{{s}_{b}}\cdot {10}^{9} & = & \frac{1}{6}\pi ({x}^{3}-{(x-{x}_{b})}^{3})\\ {\rm{z}} & = & \frac{{x}_{b}\cdot {x}_{{V}_{s}}}{2{x}_{{V}_{{s}_{b}}}}-0.5{x}_{{V}_{{s}_{b}}}\end{array}$$

where *m* is the relation between fluorescence intensity and volume of the fluorescent shell. ${F}{{S}}_{(\gamma )}^{-1}$ or ${F}{{S}}_{({b})}^{-1}$ should be input in Eq. 6 for fluorescent shells that decrease or increase in width with surface area, respectively. The relative fluorescent radius of a micro-colony is then given by Eq. 7.

7 $${I}_{x}=\frac{0.5{x}_{{V}_{{s}_{b}}}+z}{0.5x}$$

Finally, the percentage of fluorescent micro-colony volume at the steady state is given by Eq. 8, in which V_0_ is the total volume of the micro-colony and V_1_ is the non-fluorescent volume.

8 $$\begin{array}{rcl}{V}_{0} & = & \frac{1}{6}\pi {(x)}^{3}\\ {V}_{1} & = & \frac{1}{6}\pi ({(x-{I}_{x}\cdot x)}^{3})\\ {I}_{v} & = & \frac{{V}_{0}-{V}_{1}}{{V}_{0}}\end{array}$$

When micro-colonies were transferred to CM, equations described above remained valid for the *aguA::GFP* and *faeA::GFP* strains. However, the *glaA::GFP* strain was now best described by a horizontal line at *γ*, with *b* as an arbitrary intersect of this line.

### Confocal laser scanning microscopy

Fluorescence of GFP was localized in micro-colonies using a DMI 6000 CS AFC confocal microscope (Leica, Mannheim, Germany). Micro-colonies were fixed and washed (see above), transferred into a glass bottom dish (Cellview™, Greiner Bio-One, Frickenhausen, Germany, PS, 35/10 MM) and embedded in 1% low melting point agarose at 45 °C. Micro-colonies were imaged at 20x magnification (HC PL FLUOTAR L 20 × /0,40 DRY). GFP was excited by white light laser at 472 nm using 50% laser intensity (0.1 kW/cm^2^) and a pixel dwell time of 72 ns. Fluorescent light emission was detected with hybrid detectors in the range of 490–525 nm. Pinhole size was 1 Airy unit. In all cases, z-stacks were made of imaged micro-colonies using 100 slices with a slice thickness of 5.4−10 µm. Image analysis was performed with Fiji^16^.

## Supplementary information

Supplementary Material.

Supplemental Movie 1.

Supplemental Movie 2.

Supplemental Movie 3.

Supplemental Movie 4.

Supplemental Movie 5.

Supplemental Movie 6.

Supplemental Movie 7.

## Data Availability

Data are available for readers upon request.
