# Supplementary material for: Spatial induction of genes encoding secreted proteins in micro-colonies of Aspergillus niger
Source: Sci Rep. 2020 Jan 30;10:1536. doi: 10.1038/s41598-020-58535-0 (PMC6992626; doi:10.1038/s41598-020-58535-0)
Supplement: Supplementary file 1 — Supplementary Material. [file 41598_2020_58535_MOESM1_ESM.docx]

**Supplemental Material**

Spatial induction of genes encoding secreted proteins in micro-colonies of Aspergillus niger

Martin Tegelaar, David Aerts, Wieke R. Teertstra and Han A. B. Wösten

**
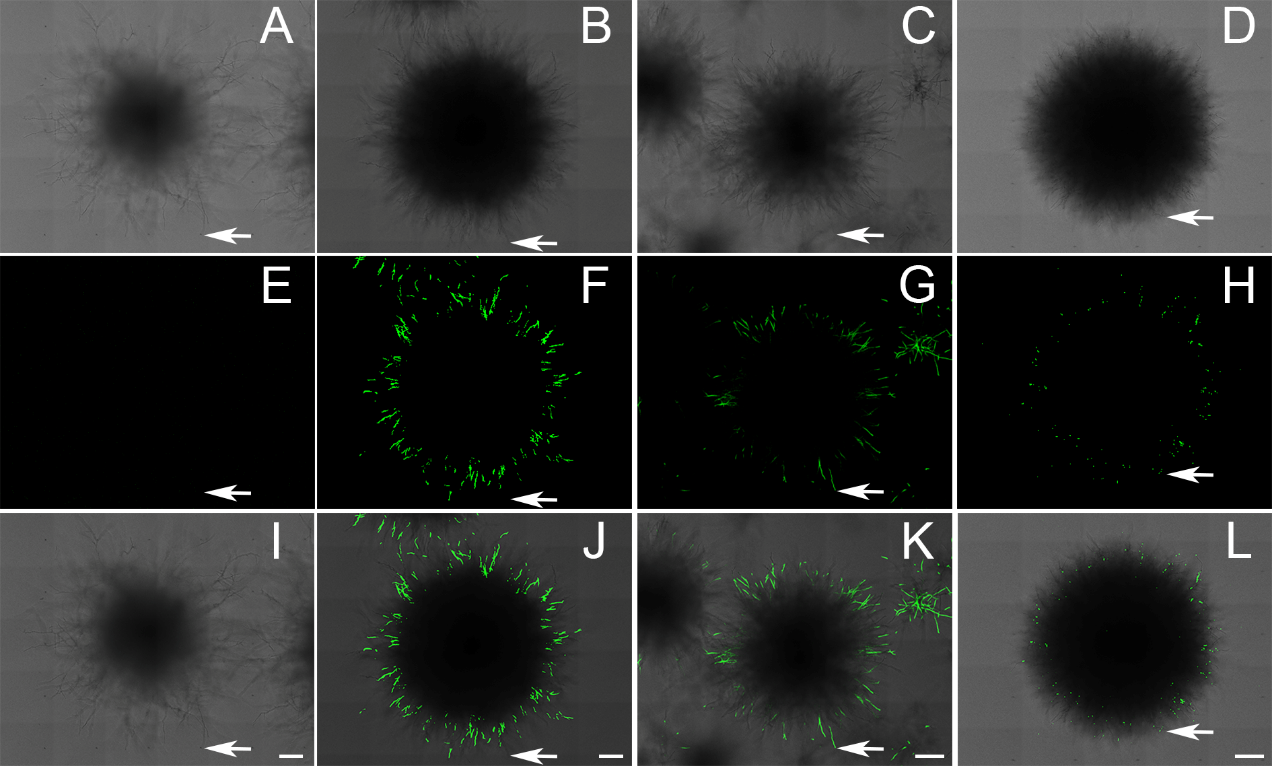
Figure S1.** Brightfield (A-D), fluorescent (E-H) and merged (I-L) images of micro-colonies of the N402 strain (A,E,I) and strains expressing gfp from the glaA (B,F,J), aguA (C,G,K) and faeA (D,H,L) promoter after transfer to inducing minimal medium. Micro-colonies of N402, glaA::GFP, aguA::GFP and faeA::GFP strains have diameters of 2140, 1989, 1357 and 1543 µm, respectively. Arrows denote the edge of the respective micro-colony. Scale bars are 200 µm.


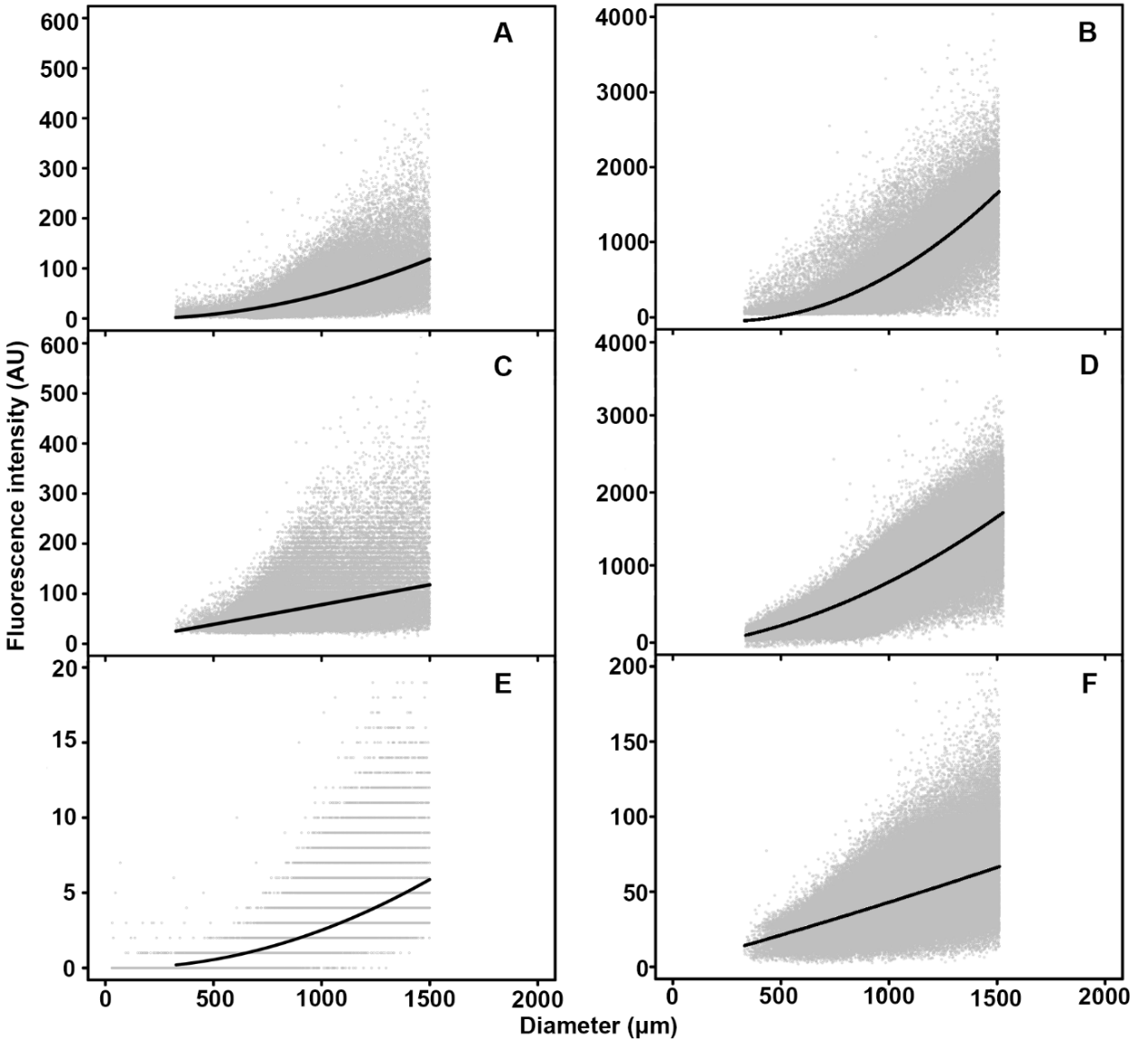
**Figure S2.** Relation between fluorescence intensity and diameter of micro-colonies of strains expressing *gfp* from the *glaA* (A,B), *aguA* (C,D) and *faeA* (E,F) promoter that had been transferred to inducing minimal medium (A,C,E) or complete medium (B,D,F). Gray circles represent individual micro-colonies. The solid line represents the best fit as determined by quantile regression of the median.


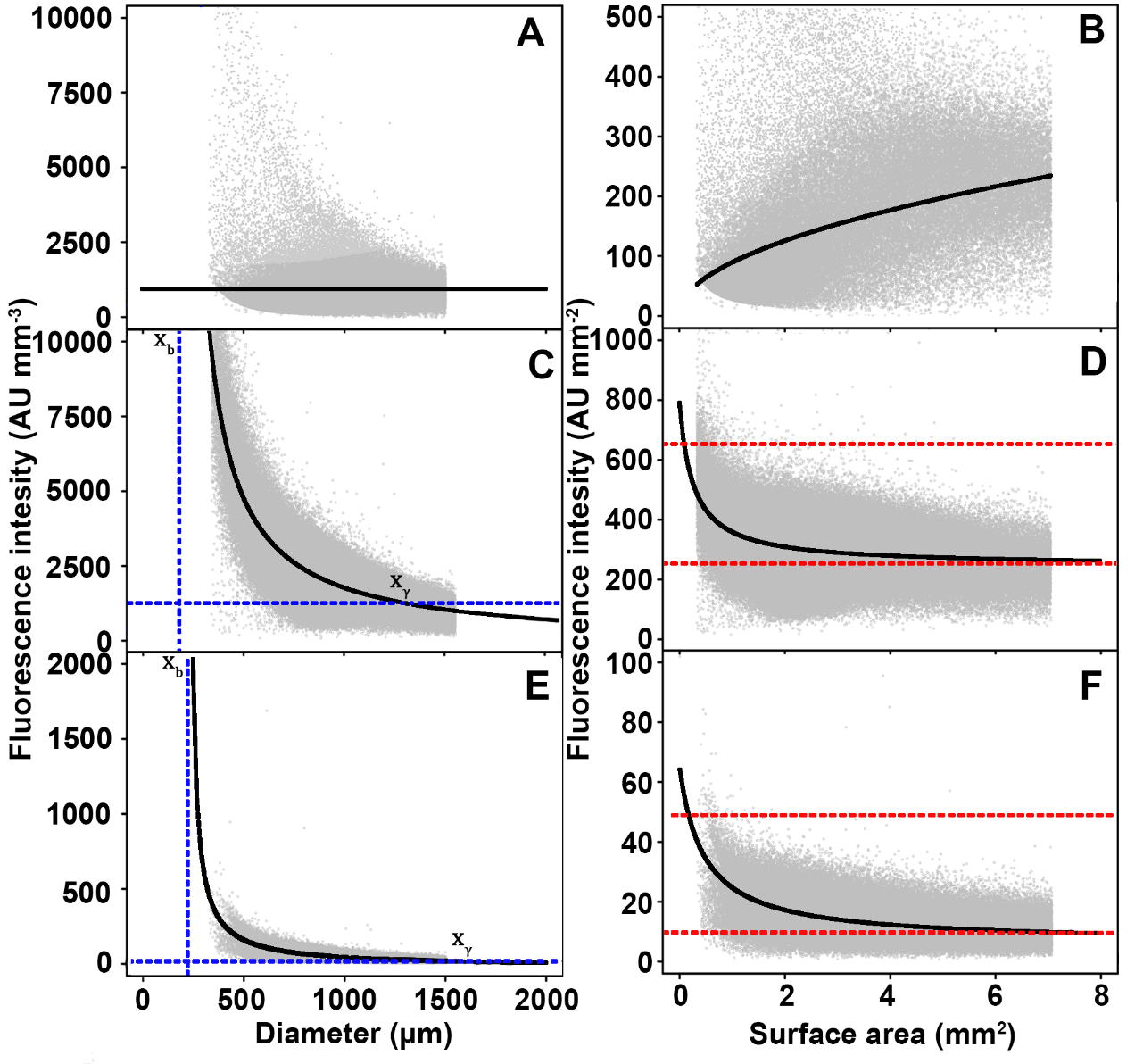


**Figure S3.** Relation between fluorescence per volume and pellet diameter (A,C,E) and fluorescence per surface area and surface area (B,D,F) of strains expressing *gfp* from the *glaA* (A, B), *aguA* (C, D) and *faeA* (E, F) promoter after transfer to inducing complete medium. Gray circles represent individual micro-colonies. Light gray squares represent individual micro-colonies discarded from the analysis using clustering with Gaussian mixture modelling. The solid line represents the best fit as determined by quantile regression of the median. Horizonal blue dashed lines represent *FV^-1^* at steady state and vertical blue dashed lines represent the diameter where fluorescence intensity is maximal (*x_b_*). The intersect between *FV^-1^* at steady state and the lower 95% confidence limit of the best fit is denoted by *x_γ_.* The red dashed lines (D-F) represent minimal and maximal fluorescence intensity mm^‑2^ as predicted by EQ3.


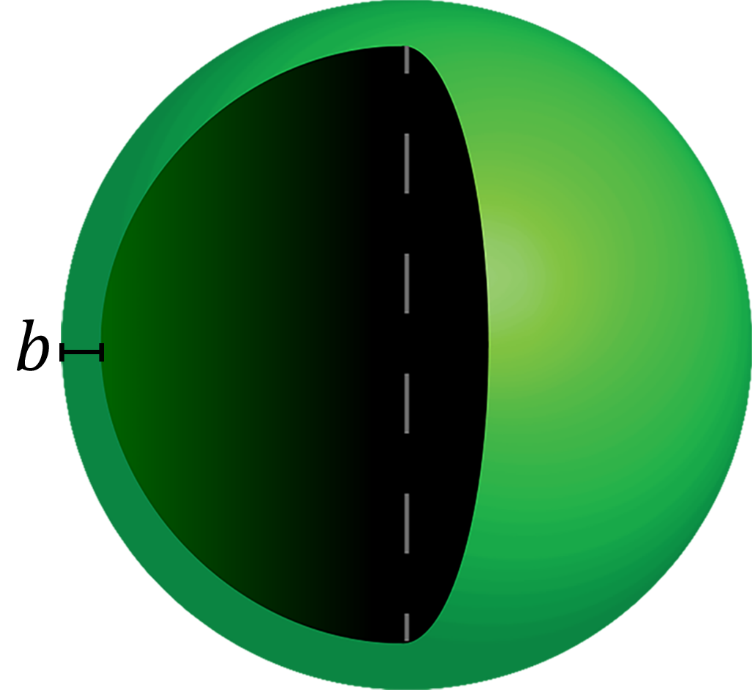


**Figure S4.** Schematic drawing of a model micro-colony as a prefect sphere. *b* denotes the width of the fluorescent shell.

**Supplemental Videos**

**Video S1.** A 3D rotating merge of a 1673 µm diameter micro-colony expressing GFP from the *glaA* promoter in a fluorescent peripheral zone.

**Video S2.** A 3D rotating merge of a 1330 µm diameter micro-colony expressing GFP from the *aguA* promoter in a fluorescent peripheral zone.

**Video S3.** A 3D rotating merge of a 1680 µm diameter micro-colony expressing GFP from the *faeA* promoter in a fluorescent peripheral zone.

**Video S4.** Z-series of a brightfield and fluorescence merge of a 2140 µm diameter N402 micro-colony (from center to top of the microcolony).

**Video S5.** Z-series of a brightfield and fluorescence merge of a 1989 µm diameter micro-colony expressing GFP from the *glaA* promoter in a fluorescent peripheral zone (from center to top of the microcolony).

**Video S6.** Z-series of a brightfield and fluorescence merge of a 1357 µm diameter micro-colony expressing GFP from the *aguA* promoter in a fluorescent peripheral zone (from center to top of the microcolony).

**Video S7.** Z-series of a brightfield and fluorescence merge of a 1543 µm diameter micro-colony expressing GFP from the *faeA* promoter in a fluorescent peripheral zone (from center to top of the microcolony).
